# The Impact of Exercises and Physical Activity Programs on Paediatric Patients Undergoing Haemodialysis in Africa: A Scoping Review

**DOI:** 10.3390/healthcare14081023

**Published:** 2026-04-13

**Authors:** Makwena Midah Sibuyi, Siyanda Alex Ngema

**Affiliations:** 1 Physiotherapy Department, School of Health Care Sciences, Sefako Makgatho Health Sciences University, Ga-Rankuwa 0208, South Africa; 2 Faculty of Science, Adelaide Tambo School of Nursing Science, Tshwane University of Technology, Pretoria 0183, South Africa; ngemasa@tut.ac.za

**Keywords:** haemodialysis, paediatric, physical activity, exercise, quality of life, scoping review, Africa

## Abstract

**Introduction**: Chronic kidney disease and kidney failure are associated with a decline in physical abilities resulting in severe health-related complications. Existing systematic reviews and meta-analyses show that exercise interventions in patients on haemodialysis enhance physical functioning, cardiovascular health, muscle strength, and overall quality of life. However, the available literature mostly stem from adult cohorts outside Africa. Thus, this scoping review aims to evaluate existing literature on the impact of exercise programs on paediatric patients undergoing haemodialysis in Africa. **Methods**: A systematic search of electronic databases, including CINAHL, EBSCO, Medline, PubMed, and Scopus, was conducted following the Arksey and O’Malley methodological framework for scoping reviews and complied with the Preferred Reporting Items for Systematic reviews and Meta-Analyses extension for Scoping Reviews (PRISMA-ScR) reporting guidelines. The inclusion criteria was applied to identify peer-reviewed articles published between 2015 and 2025, focusing on the effects, impact, and benefits of exercises and physical activity programs in paediatric patients undergoing haemodialysis aged up to 18 years. The selection process was done by two researchers pertaining to importing search results, removing duplicates, screening titles and abstracts, and analysis the reference lists of selected studies to ensure comprehensive coverage. **Results**: Two exercise-based intervention studies were eligible in the final review. In both studies, the duration of the intervention was about two months, and they included sample sizes of 60 and 50 participants. The first study, using the Paediatric Quality of Life Inventory (PedsQL-I), reported significant improvements across all dimensions in quality-of-life following muscle stretching and isometric exercises. The second study, employing the Paediatric Quality of Life Multidimensional Fatigue Scale (PedsQL-MFS) and the Depression Anxiety Stress Scale (DASS), found reductions in fatigue and psychological distress, and positive biochemical changes. A notable omission was the lack of detail regarding contraindications and precautionary measures. These are essential for informing clinical decision-making and ensuring exercises are safe. **Discussion**: The findings underscore the importance of incorporating exercise into the standard care of paediatric patients undergoing haemodialysis to facilitate better health outcomes. The fact that only two relevant studies were found highlights a narrow regional scope within Africa as both studies originated from a single country. Further research is needed to develop and implement effective exercise interventions tailored to other countries in Africa.

## 1. Introduction

Kidney failure (KF) is a significant public health challenge in Africa, with more than 50% of paediatric renal patients presenting with end-stage disease requiring renal replacement therapy (RRT) [1]. The incidence of KF in African countries is elevated due to the unavailability of resources for acute and chronic renal replacement therapy (RRT), making the prognosis unfavourable [2,3]. Chronic kidney disease (CKD) is characterised by the presence of kidney damage or an estimated glomerular filtration rate (eGFR) persisting for more than three months, and often progresses to KF requiring the need for RRT, such as dialysis or transplantation [4,5].

Dialysis provides essential support by maintaining physiological stability through the removal of metabolic waste products, regulating fluid and electrolyte balance, and maintaining acid–base homeostasis [5]. Although the causes of KF vary by age and race, the most common are congenital anomalies in children under 12 years (with 34–43% prevalence) [6].

Globally, types of dialysis used in practice include peritoneal dialysis (PD) and haemodialysis (HD) [5,6,7]. PD accounts for 9% of all RRTs and 11% of all dialysis. PD is preferred in younger children due to its technical simplicity and sustainability for home-based care [7,8,9]. Although the use of PD is attractive in low-income countries, it has limited accessibility due to the high costs of peritoneal fluids, which affects optimal usage [9].

Nonetheless, HD is commonly utilised in the adolescent group and delivered in hospital settings where there is accessibility of health professionals, including those to prescribe and monitor exercises [9]. Therefore, investigating HD is preferred to map the exercise interventions in hospital-based settings.

Children undergoing haemodialysis often report a lower health-related quality of life (QoL) compared to their peers in the earlier stages of CKD or those with a kidney transplant [10,11,12]. This decline is particularly evident across multiple domains of physical functioning, emotional well-being, social interactions, and academic performance [10,11,12]. HD is associated with children experiencing more fatigue and disruption of physical condition compared to those on PD [13]. Emotional suffering is evident regardless of the type of RRT, with both children and caregivers reporting psychological challenges of anxiety, social isolation, poor self-esteem, and lack of self-control [14]. Furthermore, school attendance and academic achievement are negatively affected due to frequent absences for treatment and hospitalisations [11].

Physical activities (PAs) and exercises are widely acknowledged as therapeutic interventions to improve physical functioning, muscle mass, and strength [15,16]. Nonetheless, children with KF undergoing haemodialysis have exercise intolerance linked to protein-energy wasting and chronic inflammation [11,17]. Although exercise during haemodialysis improves outcomes, its use in children is underutilised whilst the global clinical guidelines on type, duration, intensity, and safety are not consistent. This inconsistency restricts clinicians from promoting exercise and PAs [18,19,20]. In the African context, implementation is further limited by systemic barriers linked to inadequate dialysis infrastructure and trained multidisciplinary personnel. This scoping review aims to systematically map the evidence on exercise interventions for children undergoing haemodialysis, with a focus on Africa. It examines the types, benefits, and outcomes of such interventions to identify gaps and inform targeted programs that enhance health-related quality of life. This review answers the question: What is the scope of the literature regarding the types, impact, and outcomes of exercise interventions for children on haemodialysis in Africa?

## 2. Methods

This scoping review, which consisted of peer-reviewed published articles, was conducted in line with the framework outlined by Arksey and O’Malley [21]. The scoping review was registered on the Open Science Framework (https://doi.org/10.17605/OSF.IO/GAVNM). The registration is currently under embargo to preserve blinding during peer review.

Two researchers initiated the review, assisted by the university librarian, and developed the inclusion and exclusion criteria. The literature was systematically searched and reported following the PRISMA extension for scoping reviews (PRISMA-ScR) reporting tool and the checklist was completed [22]. The review question was formulated considering Population, Concept, and Context. Thus, this scoping review was limited to articles covering the effects of physical activities and exercise in paediatric patients undergoing HD in Africa.

### 2.1. Search Strategy

The literature search was conducted between August 2024 and April 2025 after several pilot searches. The piloted searches allowed for refinement to suit the scoping review. The following databases were searched for peer-reviewed articles related to the subject: CINAHL, MedLine, EBSCO, PubMed, and Scopus as shown in Table 1. The librarian contributed to identifying the appropriate databases, keywords, and search strings to ensure a comprehensive search. Constant communication was maintained with the librarian to ensure a smooth search process.

### 2.2. Study Selection Criteria

This scoping review included original research articles published in English in peer-reviewed journals between 2015 and 2025 (10-year period) to have a broader coverage of the literature. Studies were required to involve paediatric and adolescent participants aged 0–18 years who undergo haemodialysis treatment. Although infants and adolescents differ substantially in physiology and exercise capacity, exercise prescription within these age groups cannot be similar but customised accordingly. The research focus needed to examine PA interventions designed explicitly for children on haemodialysis. Additionally, included studies were required to report outcomes related to quality of life, physical functioning, or other health-related measures following PA interventions.

Studies were excluded from this review if they were review papers, meta-analyses, commentaries, dissertations, conference proceedings, abstracts, editorials, or book chapters, as only primary research data were considered appropriate for inclusion. Articles published in languages other than English were excluded due to resource limitations for translation and to maintain consistency in data extraction processes. Studies that did not focus specifically on populations aged 0–18 years, or those that included mixed populations without separate analysis of paediatric data, were also excluded. Furthermore, studies examining aspects other than PAs and exercises were excluded.

### 2.3. Screening and Selection Process

**Figure 1:**
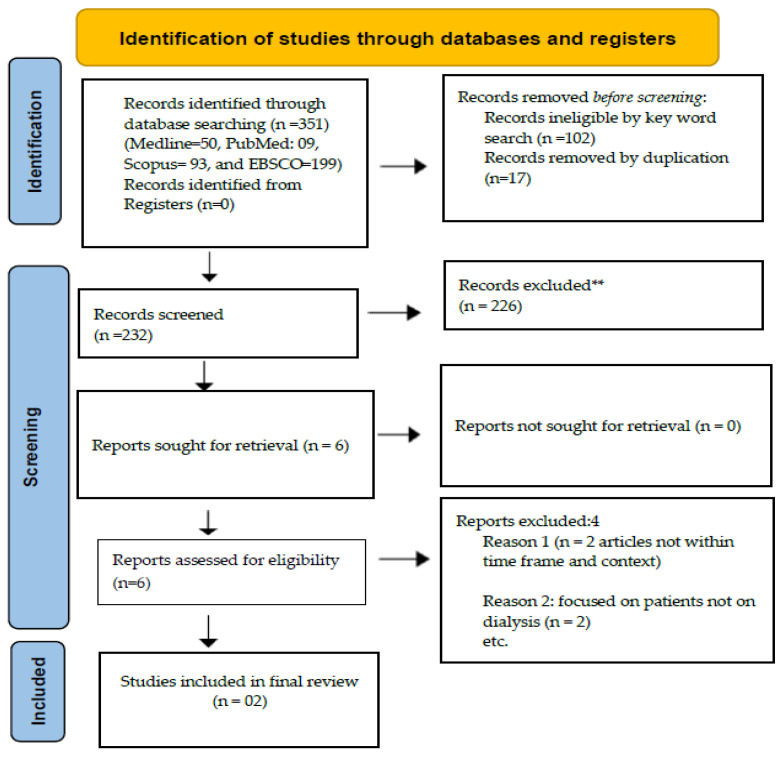
PRISMA ScR flow diagram. Source: [25]. This work is licensed under CC BY 4.0. To view a copy of this license, visit https://creativecommons.org/licenses/by/4.0/ (accessed on 12 February 2026). ** indicate how many records were excluded.

Two researchers independently screened the titles and abstracts of the identified articles to ensure rigour and reduce potential bias. Each reviewer assessed the articles using the predefined inclusion and exclusion criteria to ensure consistency and minimise bias. Disagreements pertaining to the inclusion of other types of RRT were resolved through discussion and reaching consensus between the researchers. To ensure the rigour and completeness of our search methodology, the librarian was consulted to form part of the final decision-making in the selection process and to verify the relevance, comprehensiveness, and accessibility of the selected databases and search strategies. Firstly, duplicates were removed using EndNote software version 21. Secondly, careful screening all imported studies’ titles and abstracts based on the inclusion criteria was conducted individually by the researchers. Thirdly, the reference list of the identified articles was screened to ensure comprehensive coverage of the evidence. Full-text screening was conducted for all the identified articles (see Figure 1 for the review screening process flow chart). The research team scrutinised this process.

### 2.4. Data Extraction, Analysis, and Report

A standardised data extraction sheet in Microsoft Excel was used to collate, chart, and summarise the data from studies and reports. The following basic information regarding the eligible studies was systematically extracted and tabulated to ensure consistency: authors and publication year, title of each study, country, study design, method of intervention, impact, benefits, data analysis, and key findings (see Table 1 for the basic information of the studies included in the final review).

The data extraction process was carried out systematically by two researchers who collaborated to ensure consistency and accuracy. Prior to the review, the reviewers jointly developed and finalised the data extraction table, agreeing on its contents to standardise the process (see Table 1 below for basic information on the eligible studies). Both researchers critically assessed and validated the data extraction table to align with the scoping review questions [26]. A narrative synthesis typically provides a broad overview of the existing literature, identifies vital concepts, and highlights research gaps; hence, it is best suited for this scoping review. The data analysis process was validated through multiple rounds of review by both researchers and the librarian to ensure consistency and alignment with the research objectives. Furthermore, a narrative synthesis was employed to categorise the findings [26].

## 3. Results

This scoping review included two original research articles, underscoring the scarcity of evidence in the field. Other articles were excluded as duplicates and not addressing the research question (Figure 1). The distribution of retrieved articles’ publication years, publication region, and adopted research design or methods is presented below in Table 1.

### 3.1. Geographical Location of Studies and Duration of Intervention

In this review, both studies were conducted in Egypt, highlighting a limited geographical context for the analysis. The duration of the interventions for both studies lasted two months, July to August 2022 [23], November 2020 to December 2020 [24]. One study employed a randomised control trial [23] and the other study used a quasi-experimental design [24].

### 3.2. Types of Interventions, Impact, and Outcomes

The first study investigated the effects of muscle stretching and isometric exercises on the quality of life (QoL) of children undergoing HD [23]. The Paediatric Quality of Life scale version 4.0 (PedsQL™), translated into Arabic, was the outcome measure used to assess health-related QoL before and after the exercises. The PedsQL™ consisted of 23 items divided into four domains: physical, emotional, social functioning, and school performance. Initially, there were no significant differences between the study and control groups across these domains. However, post-intervention results showed that the study group experienced significant improvements in the total PedsQL™ score compared to the control group (*p* = 0.001), with an effect size of 0.531 indicating a moderate-to-large difference. Notable increases were observed in physical function (from 132.50 to 483.33), emotional functioning (from 141.67 to 356.67), social functioning (from 195.83 to 419.17), and school performance (from 165.0 to 389.17). PedsQL™ scores are typically reported as averages (0–100), but in this study they were summed to highlight total improvements across items. After two months of intervention, 66.7% of participants reported improved QoL, compared to just 3.3% in the control group. No significant correlation was found between child age and total QoL (*p* = 0.47) [23].

The second study assessed the impact of intradialytic exercise on fatigue, psychological distress, and biochemical markers [24]. Fatigue symptoms were measured using the Paediatric Quality of Life Inventory Multidimensional Fatigue Scale (PedsQL-MFS), consisting of three subscales: sleep/rest fatigue, cognitive fatigue, and general fatigue. Psychological distress was evaluated with the Depression Anxiety and Stress Scale (DASS-21). Researchers also measured biochemical markers through blood samples for creatinine, blood urea nitrogen (BUN), sodium, calcium, potassium, and phosphorus, comparing the results monthly after dialysis.

Key findings included no significant differences in the total mean scores of depression, anxiety, and stress between study and control groups before the exercise program (*p* = 0.856) [24]. However, significant differences were observed after 4 and 8 weeks of intervention (*p* = 0.000). The study group showed improvements in BUN, calcium, and phosphorus levels compared to the pre-intervention levels. There was a significant difference in total chemical values between groups (*p* = 0.001). Additionally, a positive correlation existed between fatigue scores and anxiety, depression, and stress in the study group, with a highly significant difference compared to the control group (*p* < 0.001) [24].

## 4. Discussion

This scoping review provided insight into the extent of evidence regarding the types, benefits, and impact of exercise interventions for children on haemodialysis in Africa. Two studies met the inclusion criteria and were conducted between 2020 and 2022 [23,24]. Both studies demonstrated that exercise interventions are feasible in this population and reported improvements in physical function and quality of life. These findings provide preliminary evidence that exercise may be beneficial for paediatric patients on haemodialysis in African settings, though the evidence base remains limited.

The scarcity of studies in this context contrasts with the broader literature on exercise in patients with kidney disease. For example, systematic reviews and meta-analyses in adult populations have shown that home-based exercise interventions can improve physical performance in patients undergoing maintenance dialysis [27]. Similarly, studies in children with CKD not on dialysis, such as those conducted in Egypt, have reported improvements in quality of life and functional capacity following progressive resistance exercise [28]. While these findings highlight the potential relevance of exercise interventions, they were conducted in different populations and settings and, thus, cannot be directly applied to the African context.

Other approaches to monitoring and promoting physical activity, such as pedometers [29,30], validated questionnaires such as the Global Physical Activity Questionnaire (GPAQ) [31], and technological innovations including virtual reality gaming [32,33,34,35,36], have been explored in adult or non-African populations. These studies provide useful context but remain outside the scope of the included evidence.

Nonetheless, the two included studies provide early indications that exercise interventions may improve outcomes for children on haemodialysis in Africa. However, the limited number of studies underscores the need for further research to establish the effectiveness and sustainability of such interventions in this specific population.

### Recommendations

To enhance the quality of life for paediatric patients undergoing HD, it is essential to develop standardised guidelines for physical exercise that cater specifically to their needs. Personalised exercise plans, created in collaboration with healthcare professionals, should be integrated into routine dialysis treatments. Educating healthcare providers and engaging families will foster a supportive environment, while the use of technology, such as mobile apps, can help monitor activity levels and motivate children to be more active. Additionally, implementing multi-disciplinary approaches, and encouraging peer support groups will create a holistic framework that promotes physical activity. This comprehensive strategy aims to improve not only the physical well-being but also the emotional and social aspects of children on dialysis, particularly in underrepresented regions like Africa. As these recommendations are expert-informed rather than evidence-based, future studies should incorporate clearly defined safety parameters, including patient selection criteria, monitoring guidelines, and response plans for exercise-related complications. Doing so will enhance clinical utility and support the development of standardised, evidence-based rehabilitation frameworks across diverse care settings.

## 5. Limitations

Despite the generally positive outcomes reported across studies, several limitations were apparent. Firstly, the heterogeneity in intervention designs, outcome measures, and evaluation timeframes makes direct comparisons challenging. Secondly, both studies employed unique designs over different intervals, potentially limiting the strength of evidence. Thirdly, the long-term sustainability of improvements was not measured, with most studies focusing on short-term outcomes. No mention was made of barriers linked to exercise interventions. This observation highlights a potential gap in addressing contraindications and precautions that should be considered when implementing these exercise programs. It is crucial to recognise that while exercise can offer numerous benefits, there may be specific circumstances or health conditions that warrant caution, emphasising the importance of individualised approaches to exercise. The selection criteria excluded studies written in languages other than English and sources such as theses, dissertations, and conference proceedings. Thus, this approach could have introduced selection bias and restricted the extent of the findings, especially in the African context, as dissemination of data in high-impact journals may be limited.

Lastly, both studies in this review were conducted in a single country (Egypt), for which the situation in other regions and or countries may differ.

## 6. Conclusions

This scoping review identified only two studies, and both reported positive outcomes, including improvements in functional capacity and quality of life. These findings provide preliminary evidence that exercise interventions may be feasible and beneficial in paediatric patients on haemodialysis. However, the evidence base is limited, and should be interpreted with caution. It is not possible to determine the broader applicability of these interventions across diverse African contexts. The findings highlight the need for multicentre studies conducted in different countries, using rigorous methodologies and longer-term follow-up, to establish the effectiveness, safety, and sustainability of exercise interventions for paediatric haemodialysis patients in Africa.

## 7. Contribution of the Review

This scoping review contributes to physiotherapy and paediatric nephrology by examining the benefits of exercise and physical activities in HD, comprehensively analysing the existing literature on the outcomes of exercise and physical activities among paediatric patients in haemodialysis settings. It highlights the need for more studies on this phenomenon in various countries within the African region. Furthermore, the review paves the way for future studies that include the roles of physiotherapy, families or caregivers, and support of other multidisciplinary team members in promoting exercises and physical activities in paediatric patients undergoing HD.

## Figures and Tables

**Table 1:** Articles for the final review.

Authors and Year of Publication	Title of the Study	Objective	Country	Population Sample and Size	Study Design	Exercise Intervention Method	Key Findings and Gaps Identified
Khalf-Allah et al., 2024 [23]	Effect of muscle stretching and isometric exercises on quality of life in children undergoing regular haemodialysis	The study aimed to investigate the effect of muscle stretching and isometric exercises on QoL of children undergoing haemodialysis.	Egypt	68 children from a total population of 87 children undergoing haemodialysis at the Paediatric Nephrology Unit in Assiut University Children’s Hospital aged from 6 to 18 years old. The sample included 60 children with half split to either study group or control group by a web-based randomizer.	Randomised control trial.	A 40 min program conducted 3 times weekly for 2 months during haemodialysis sessions. Stretching exercises (20 min, 2nd hour): targeted calf, hamstring, and quadriceps muscles with 10–15 repetitions to improve flexibility. Isometric exercises (20 min, 3rd hour): static contractions of chest, biceps, stomach, and hip muscles held for 3–5 s, 10–15 repetitions each. Safety protocols avoided body parts connected to dialysis machines. Children received initial supervision from a trained researcher, progressing to independent performance under supervision with gradual repetition increases based on individual tolerance.	Results showed 66.7% of exercising children achieved good quality of life compared to only 3.3% in the control group. All domains improved significantly: physical functioning increased nearly four-fold, while emotional, social, and school performance also enhanced substantially. The program benefited all age groups equally, demonstrating that structured exercise during dialysis can transform the overall well-being of paediatric patients with kidney disease.
2. Salama et al., 2022 [24]	Effect of intradialytic exercise on fatigue, psychological distress, and biochemical findings among haemodialysis children.	To evaluate the effectiveness of intradialytic exercise on fatigue, psychological distress, and biochemical findings among haemodialysis children.	Egypt	50 children aged 6–18 years with chronic renal disease on haemodialysis. Using a simple random sample, half were split into either control or study group.	Quasi-experimental design	A quasi-experimental design was used to conduct the study and two groups (study and control group pre/post-test) were used to achieve the study purpose. Study group: they practiced exercises for two months, three times a week, during the first two hours of haemodialysis sessions. Control group: they only received scheduled treatment.	The study concluded that intradialytic exercise had a positive effect on fatigue, psychological distress, and biochemical findings among haemodialysis children

## Data Availability

No new data were created or analysed in this study.

## Abbreviations

CKD	Chronic Kidney Disease
KF	Kidney Failure
RRT	Renal Replacement Therapy
eGFR	Estimated Glomerular Filtration Rate
PD	Peritoneal Dialysis
HD	Haemodialysis
QoL	Quality of Life
PA	Physical Activity
